# Prognostic Value and Sex-Related Differences in Chest Pain in Patients with Acute Pulmonary Embolism: A Prospective Cohort Study Beyond Myocardial Ischemia

**DOI:** 10.3390/jcm15072732

**Published:** 2026-04-04

**Authors:** David Frumkin, Marie C. Merten, Ann-Sophie Eggers, Regina Stegherr, Marieke Lankeit, Markus H. Lerchbaumer, Gerhardt Hindricks, Gerd Hasenfuß, Stavros Konstantinidis, Matthias Ebner

**Affiliations:** 1 Deutsches Herzzentrum der Charité, Department of Cardiology, Angiology and Intensive Care Medicine, Campus Charité Mitte, Charitéplatz 1, 10117 Berlin, Germany; david.frumkin@dhzc-charite.de (D.F.);; 2 Charité—Universitätsmedizin Berlin, Corporate Member of Freie Universität Berlin and Humboldt-Universität zu Berlin, Charitéplatz 1, 10117 Berlin, Germany; 3 DZHK (German Centre for Cardiovascular Research), Partner Site Berlin, 10785 Berlin, Germany; 4 Clinic of Cardiology and Pneumology, University Medical Center Göttingen, 37075 Göttingen, Germany; 5 Deutsches Herzzentrum der Charité, Department of Cardiology, Angiology and Intensive Care Medicine, Campus Charité Wedding, Augustenburger Platz 1, 13353 Berlin, Germany; 6 Institute of Biometry and Clinical Epidemiology, Charité—Universitätsmedizin Berlin, Corporate Member of Freie Universität Berlin and Humboldt-Universität zu Berlin, Charitéplatz 1, 10117 Berlin, Germany; 7 Center for Thrombosis and Hemostasis (CTH), University Medical Center Mainz, 55131 Mainz, Germany; 8 Department of Radiology, Campus Charité Mitte (CCM), Charité—Universitätsmedizin Berlin, Corporate Member of Freie Universität Berlin and Humboldt-Universität zu Berlin, Charitéplatz 1, 10117 Berlin, Germany; 9 DZHK (German Centre for Cardiovascular Research), Partner Site Göttingen, 37075 Göttingen, Germany; 10 Department of Cardiology, Democritus University of Thrace, 68100 Alexandroupolis, Greece; 11 Department of Cardiology, Klinik Ottakring, Montleartstraße 37, 1160 Vienna, Austria

**Keywords:** pulmonary embolism, chest pain

## Abstract

**Background:** While previous studies indicate an association between chest pain and favorable clinical outcomes in patients with pulmonary embolism (PE), the extent and underlying mechanisms of this effect remain inadequately defined. **Methods**: We investigated the prognostic value of chest pain with regard to in-hospital adverse outcomes and the association of chest pain with age and sex in consecutive patients with confirmed PE enrolled in a single-center registry between 2008 and 2019. **Results**: Of 858 patients (52% female) included in this study, 435 (51%) had chest pain at presentation. Chest pain was more prevalent in younger individuals aged 18–34 years (74%) compared to patients >34 years (46%). The prevalence of coronary artery disease (CAD) was similar in patients with and without chest pain (17.0% vs. 16.1%). Chest pain patients less frequently presented with elevated troponin levels (*p* < 0.001) or signs of right heart strain (RHS; *p* = 0.007) but more frequently exhibited imaging signs of pulmonary infarction (*p* = 0.001). Chest pain was associated with lower risk of adverse outcome (OR 0.35 [95% CI: 0.19–0.65]) and in-hospital mortality (OR 0.31 [95% CI: 0.13–0.74]). Multivariable models confirmed a prognostic effect independent of sex, comorbidities and results of risk stratification algorithms. **Conclusions**: In acute PE, chest pain is a favorable prognostic marker irrespective of sex. Chest pain patients are less likely to suffer from myocardial ischemia or show signs of RHS but more frequently show imaging signs of pulmonary infarction, suggesting pleuritic irritation rather than myocardial ischemia as the likely cause of pain.

## 1. Introduction

Acute pulmonary embolism (PE) remains a frequent and life-threatening disease associated with significant morbidity and mortality [1,2,3,4]. Beyond pre-existing comorbidities, the occurrence and severity of acute right heart failure, resulting from partial or complete obstruction of the pulmonary arteries, is a critical determinant of early mortality. Various risk stratification algorithms are employed to assess the risk of complications and mortality, guiding the clinical management of patients with PE [5].

In addition to dyspnea, syncope, and hemoptysis, chest pain is a common symptom of PE, reported in approximately 50–70% of patients [6,7,8,9]. To date, the prognostic significance of chest pain in patients with PE has been incompletely elucidated. A retrospective cohort study of 1306 patients with PE demonstrated that those presenting with chest pain were younger and exhibited lower early (in-hospital) and six-month mortality rates compared to those without chest pain [9]. However, data on troponin levels as a marker of myocardial ischemia were not reported, and data on signs of right heart strain (RHS) on diagnostic imaging was available for only 39% of patients, thereby limiting the assessment of baseline mortality risk. Further evidence suggesting an association between chest pain and good prognosis was provided by an analysis of 4145 PE patients from a large non-consecutive multinational registry [6]. This analysis revealed that a subgroup of patients with clinically defined pulmonary infarction (presence of pleuritic chest pain or hemoptysis) had a more favorable prognosis than patients with isolated dyspnea or circulatory collapse. However, it is important to note that thoracic pain was not independently examined for its prognostic value in this analysis, precluding definitive conclusions regarding its specific impact.

Furthermore, even though PE affects men and women in equal proportions and important sex specific differences have been demonstrated in other fields of cardiovascular medicine, e.g., in stroke [10] or coronary artery disease [11], neither of the available studies investigated possible effects of sex on the prognostic effect of chest pain.

This prospective cohort study evaluated the prognostic significance of chest pain in patients with PE, with particular attention to potential sex-related differences and its association with myocardial injury and pulmonary infarction.

## 2. Methods

### 2.1. Pulmonary Embolism Registry Göttingen

The Pulmonary Embolism Registry of Göttingen (PERGO) prospectively included consecutive patients with objectively confirmed acute PE aged ≥18 years admitted to the University Medical Center Göttingen, Germany. The study protocol has been described in detail previously [12,13,14]. Briefly, patient recruitment is performed by daily screening of new admissions to the emergency department and reports of computed tomography pulmonary angiographies (CTPA) performed. After obtaining informed consent for participation in PERGO, data on comorbidities, previous medication, symptoms, results of diagnostic tests, treatment, and clinical course are recorded using a standardized case report form. The modes of data collection did not change over the years.

The present analysis included patients enrolled in PERGO between September 2008 and September 2019. We excluded patients with (1) another acute cardiorespiratory illness, such as acute myocardial infarction, left-heart decompensation, or respiratory decompensation responsible for clinical presentation and symptoms; (2) recurrent PE (only the first event was included in the analysis); (3) cardiopulmonary resuscitation and/or intubation at the time of PE; (4) asymptomatic PE diagnosed as an incidental finding during diagnostic work-up for another suspected disease; or (5) lack of data on the presence of chest pain.

Diagnostic and therapeutic management was in accordance with the European Society of Cardiology (ESC) 2008 (09/2008–08/2014) and 2014 (09/2014–09/2019) guidelines [15,16] and local standard operating procedures. All related decisions were left to the discretion of the treating physicians and not influenced by the study protocol. This study was conducted in accordance with the amended Declaration of Helsinki and was approved by the local independent Ethic Committee of the Medical University Göttingen, Germany (application number: 14/6/10); all patients gave informed written consent for participation in this study.

Right heart strain by CTPA was defined as right-to-left ventricular (RV/LV) diameter ratio ≥ 1.0. Thrombus-associated parenchymal changes were defined as pulmonary opacification adjacent to the territory of the occluded pulmonary artery branches on CTPA.

Laboratory parameters were measured from blood samples obtained as part of routine clinical management at the time of PE diagnosis. Plasma concentrations of troponin T or I were determined using high-sensitivity assays (hsTnT; Roche Diagnostics, Mannheim, Germany; hsTnI: ARCHITECT stat hsTnI assay, Abbott Laboratories, Chicago, IL, USA) and relevant increases were defined as hsTnT ≥ 14 pg/mL and hsTnI > 16 pg/mL [17,18].

### 2.2. Definition of Endpoints

Occurrence of PE-related complications (defined as PE-related death, cardiopulmonary resuscitation or catecholamine treatment) during hospitalization was selected as the primary endpoint. The secondary endpoint was all-cause mortality during the in-hospital stay.

### 2.3. Statistical Analysis

A descriptive analysis of the total patient population was performed. For this purpose, categorical variables were specified as absolute numbers or percentages and compared using the Fisher exact test/χ2 test. Continuous variables are expressed with mean and standard deviation and compared using two-sample Welch-t-test in case of normal distribution. Variables not following a normal distribution are expressed as median and interquartile range (IQR) and compared via Mann–Whitney U test.

The study cohort was stratified and compared based on the presence of chest pain on admission, age groups, sex and thrombus-associated parenchymal changes on diagnostic imaging.

Univariable logistic regression analyses were performed to assess the prognostic relevance of variables with regard to the study endpoints. The results are reported as odds ratio (OR) with the corresponding 95% confidence interval (CI). To further investigate the predictive value of chest pain for PE-related complications during hospitalization and in-hospital mortality, multivariable logistic regression analyses were performed: In Model I chest pain was adjusted for symptoms identified as prognostically relevant for the respective endpoint in univariate analysis. In Model II, we conducted a multivariable analysis that adjusted for the presence of coronary artery disease (CAD). In Model III chest pain was adjusted for comorbidities identified as prognosis-relevant for the respective endpoint in univariate analysis, while Model IV corrected for baseline mortality risk assessed using the ESC 2019 algorithm (low risk, intermediate-low risk, intermediate-high risk, high risk). Model V included chest pain and sex as independent variables.

To identify the optimal age cut-offs for predicting the occurrence of chest pain, we conducted a logistic regression model including a B-spline with four degrees of freedom and represented the results using splines. This allowed us to establish optimal age categories for logistic regression analyses.

A two-sided significance level of α = 0.05 is defined to show statistical significance. The statistical analysis was performed using the R statistical software environment (R Foundation for Statistical Computing, Vienna, Austria, http://www.R-project.org (accessed on 15 July 2025), version 3.6.3) and IBM SPSS (Version 30.0).

During the preparation of this work the first author used artificial intelligence software (DeepL. (2025). DeepL Translator, https://www.deepl.com/ and OpenAI. (2025), ChatGPT (May 31 version) [large language model], https://chat.openai.com/) in order to enhance readability and create tables and graphics. After using these tools, all authors reviewed and edited the text and tables/figures as needed and take full responsibility for the content of the publication.

## 3. Results

### 3.1. Study Population

**Figure 1:**
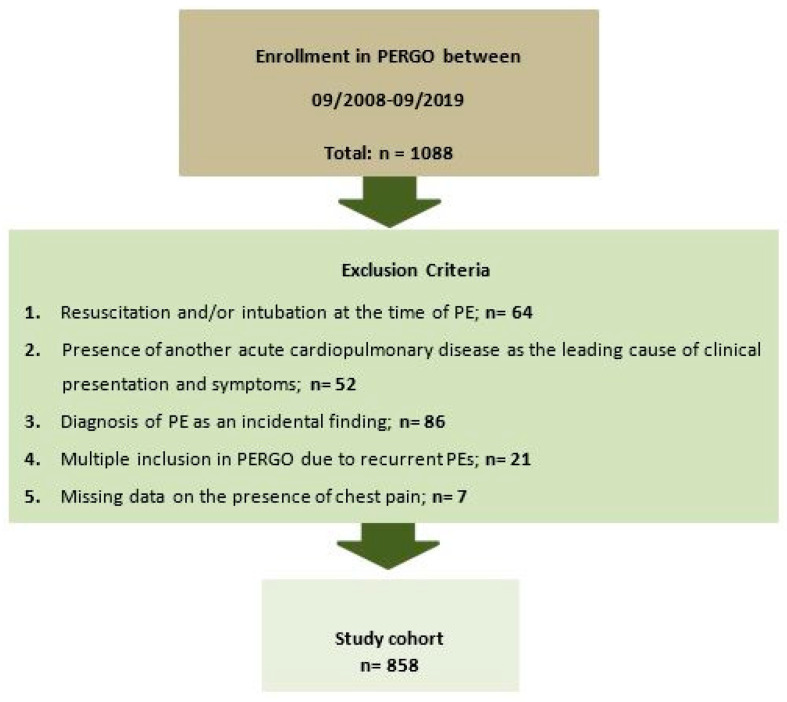
Flowchart of study population composition after application of exclusion criteria.

Of 1088 patients enrolled in PERGO between September 2008 and September 2019, 230 met one of the study’s exclusion criteria (Figure 1).

Therefore, a total of 858 patients were included in this study. The study population consisted of 446 women (52.0%) and 412 men (48.0%). Median age of the total population was 68 (56–78) years. Chest pain was reported at the time of PE diagnosis by 50.6% of patients. In the overall cohort, 5.9% of patients developed PE-related complications, and 3.3% of patients died during hospitalization. Further characteristics are shown in Table 1.

Table 1 shows baseline characteristics and outcomes stratified according to the presence of chest pain at the time of PE diagnosis. Patients with chest pain were four years younger than patients without chest pain (*p* = 0.001). There was no difference in sex distribution.

There was no significant difference in the prevalence of CAD between patients with and without chest pain (17.0% vs. 16.1%). Other chronic cardiovascular diseases such as arterial hypertension or atrial fibrillation were less common in patients with chest pain (60.9% vs. 68.2% and 8.0% vs. 12.8%). A similar trend was observed for chronic heart failure (CHF; 12.0% vs. 16.5%). Cancer-associated PE was also less frequent in patients with chest pain (19.1% vs. 26.7%).

**Figure 2:**
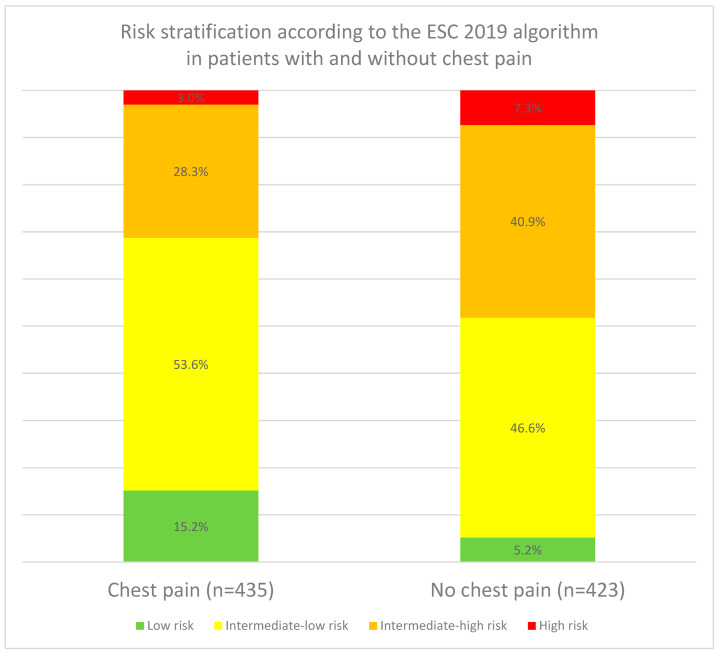
Risk of early mortality in patients stratified by the occurrence of chest pain. ESC = European Society of Cardiology.

Prognostically unfavorable symptoms such as concomitant syncope or altered mental status occurred significantly less frequently in patients with chest pain (*p* < 0.001 for both). In addition, patients with chest pain were less likely to present with hypoxia (*p* < 0.001), tachycardia (*p* < 0.001) or hypotension (*p* = 0.014). Furthermore, imaging signs of RHS (*p* = 0.007) or laboratory signs of myocardial damage (elevated troponin level; *p* < 0.001) were less frequently observed. Hence, patients with chest pain were more frequently assigned to a lower risk class according to the ESC 2019 algorithm than patients without chest pain (*p* < 0.001, Figure 2).

Importantly, lung parenchymal changes on diagnostic imaging were more frequently detected in patients with chest pain (41.8% vs. 29.5%, *p* = 0.001). In addition, hemoptysis was more common in patients with chest pain (4.1% vs. 1.4%, *p* = 0.016), and patients with chest pain were more likely to require antibiotic therapy due to pneumonia during the in-hospital stay (42.3% vs. 29.6%, *p* = 0.001).

### 3.2. Influence of Age on the Occurrence of Chest Pain

**Figure A1:**
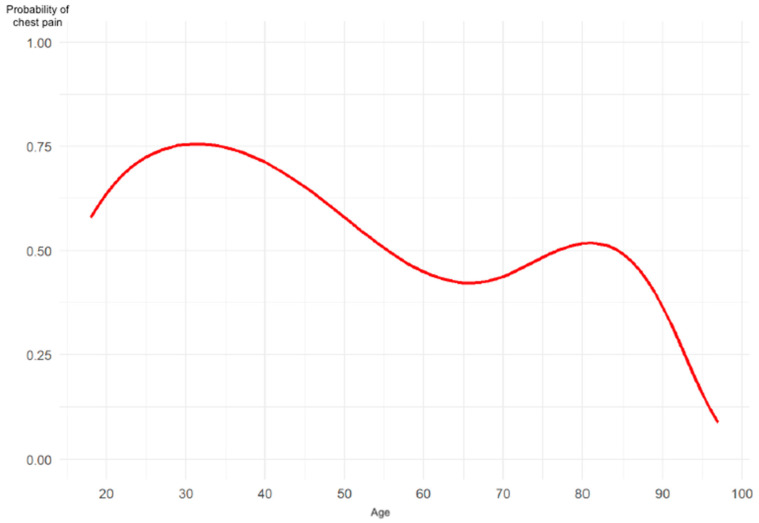
Results of logistic regression modeling the effect of age on the occurrence of chest pain with a B-spline showing the inflection points used for later age categorization.

Due to the finding that chest pain was associated with younger age, we conducted a logistic regression model. Figure A1 shows the subsequent analysis illustrating the results of logistic regression modeling the effect of age in more detail with a B-spline using four degrees of freedom and identified inflection points. For optimal interpretability in the clinical setting, we used the identified inflection points to categorize the cohort into age groups (18–34, 35–59, 60–79, ≥80 years) and calculated the OR for chest pain for each group (presented in Table 2).

The presence of chest pain was significantly associated with young age (*p* < 0.001). The youngest age category (18–34 years) showed an OR of 2.88 for the occurrence of chest pain (Table 2). In addition, an age dependence was observed in the majority of study variables associated with the occurrence of chest pain (Table 3). Chest pain as well as the detection of thrombus-associated parenchymal changes and the clinical diagnosis of pneumonia most likely occurred in the youngest age group (18–34 years) and were less frequently observed with increasing age.

In the study cohort, the occurrence of chest pain was associated with a reduced risk of PE-related complications (3.2% vs. 8.7%, OR 0.35 [95% CI: 0.19–0.65]) and all-cause mortality (1.6% vs. 5.0%, OR 0.31 [95% CI: 0.13–0.74]) during hospitalization. Further results of univariate logistic regression analyses are presented in Table A1.

To explore the prognostic effect of chest pain, the overall cohort was divided at the median inflection point derived from our logistic regression model (<60 and ≥60 years) (Table 4). In individuals under 60 years, chest pain was found to have a favorable odds ratio against both PE-related complications and all-cause mortality during hospitalization. In patients ≥60 years, there was a beneficial prognostic effect with regard to PE-related complications; however, this effect was not observed for all-cause mortality.

The prognostic significance of chest pain was further investigated using several multivariable logistic analyses. Model I showed that the prognostic value of chest pain with regard to PE-related complications remained independent of other prognostically relevant symptoms identified in the univariate analysis (OR 0.42 [95% CI: 0.22–0.80]).

Furthermore, the prognostic effect of chest pain with regard to PE-related complications was independent of pre-existing CAD (Model II: OR 0.34 [95%CI: 0.18–0.64]) and other prognostically relevant comorbidities (Model III: OR 0.41 [95% CI: 0.22–0.78]). Similar results were observed for all-cause mortality. Finally, the prognostic value of chest pain was independent of ESC 2019 risk class (Model IV) and patient sex (Model V; for all results of multivariable logistic analysis models see Table A2).

## 4. Discussion

In the present study, we evaluated the prognostic value and sex-related differences in chest pain in patients with acute PE. Our findings in a prospective cohort study of 858 patients can be summarized as follows: Chest pain is a favorable prognostic marker in patients with acute PE and was associated with a lower risk of PE-related complications and all-cause mortality during hospitalization. Patients with chest pain were younger, had fewer comorbidities and more frequently showed thrombus-associated parenchymal changes indicative of possible pulmonary infarction. Chest pain was evenly distributed between females and males, and its prognostic value was independent of sex.

In our cohort, chest pain was reported as a symptom on admission in 50.6% of PE patients. This finding is in line with other studies that observed the occurrence of chest pain in 49% [19] to 59% [9] of patients with acute PE. Importantly, patients who reported chest pain less frequently had elevated troponin levels at presentation compared to non-chest pain patients. This makes an ischemic cause of the pain unlikely.

Another pathophysiological explanation for chest pain in the context of PE is the occurrence of pleuritic irritation due to pulmonary infarction. The first descriptions of pulmonary infarction in acute PE from Hampton and Castleman in 1940 demonstrated an incidence of up to 70% in patients with acute PE, both radiographically and confirmed by autopsy in a study of 370 patients [20]. Dalen et al. described that patients with pulmonary infarction usually present with pleuritic pain and/or hemoptysis [21]. Different from these early reports, more recent studies report a lower incidence (16.9–50%) [6,22,23]. Contemporary publications provide evidence that patients who developed pulmonary infarction in the setting of acute PE are younger than patients without pulmonary infarction [6,19,22] and have a significantly lower prevalence of cardiovascular comorbidities [22,23]. Current pathophysiological considerations assume that, after the thrombotic occlusion of pulmonary arteries, the bronchial arteries are recruited as the primary source of lung perfusion. The resulting elevated blood pressure in the bronchial circulation causes an increase in capillary blood flow, which favors extravasation of erythrocytes (alveolar hemorrhage). Failure to resorb the bleeding leads to tissue necrosis resulting in infarction [24]. Preexisting cardiopulmonary conditions may “protect” against pulmonary infarction by increasing ischemic tolerance via the development of collateral vessels (23), thus explaining the lower incidence in older PE patients with chronic comorbidities.

Several findings in our cohort support the assumption that chest pain in the setting of acute PE may indeed be caused by pleuritic irritation due to pulmonary infarction and/or concomitant pneumonia: Chest pain patients more frequently reported hemoptysis and thrombus-associated parenchymal changes on diagnostic CT imaging as a possible imaging correlate of pulmonary infarction. In accordance with these radiological findings, patients with chest pain more frequently received antibiotic treatment for clinically diagnosed pneumonia compared to patients without chest pain. Finally, similar to reports observing pulmonary infarction more frequently in younger individuals with fewer comorbidities [22], we observed a higher frequency of chest pain in this patient group.

Furthermore, our results confirm prior reports that observed a favorable prognostic value of chest pain in patients with acute PE [6,9]. Wong et al. described the prognostic effect to be independent of Simplified Pulmonary Embolism Severity Index (sPESI) class, the Charlson Comorbidity Index score, patient age, underlying malignant disease and vital parameters [9]. In agreement with these results, the prognostic value of chest pain in our study was independent of other outcome predictors such as altered mental status, syncope or relevant comorbidities in different models of multivariable analyses. In addition, our results show that chest pain was equally often present in men and women, and a multivariate analysis confirms the prognostic value to be independent of sex (OR 0.35 [95% CI: 0.18–0.66]).

While information on RHS and troponin levels was largely lacking in the earlier study by Wong et al., the availability of these important risk markers in our data set enabled us to further investigate PE severity in patients with and without chest pain: Our analyses show that chest pain was associated with lower rates of RHS and myocardial ischemia and lower in-hospital mortality risk. However, while the lower estimated baseline mortality risk may partially account for the observed beneficial prognostic value of chest pain, our multivariable regression analyses demonstrate that adjustment for the ESC 2019 risk class only modestly reduced the prognostic information provided by the presence of chest pain on admission (OR: 0.49 [95% CI: 0.26–0.95]), making additional contributing factors likely.

One possible explanation may be found in the baseline characteristics of patients in whom chest pain occurred: Concomitant malignancy is a major driver of short-term adverse outcome and mortality in patients with acute PE [14,25]. In our cohort, patients with cancer less often reported chest pain on admission (19.1% vs. 26.7%, *p* = 0.008). This finding is in accordance with a report by Au et al. (26), who also observed a lower incidence of chest pain in PE patients with cancer compared to those without known malignancy (18.2% vs. 37.4%, *p* < 0.01). Furthermore, in our cohort, other chronic conditions such as arterial hypertension and anemia were also less frequent in patients with chest pain. A similar trend was seen for CHF.

In contrast, in the present study, a clear correlation was observed between younger age and the presence of chest pain. Chest pain most frequently occurred in patients aged 18 to 34 years (OR: 2.88 [95% CI: 1.69–5.14]), and the relative frequency decreased with increasing age. This finding is in line with earlier reports observing that patients with chest pain in median were 8 years younger compared to patients without chest pain [9].

Age likely influences PE symptoms due to preexisting comorbidities. Unsurprisingly, in our cohort, older patients more frequently suffered from cardiopulmonary comorbidities such as CAD or CHF. Older patients were also more frequently diagnosed with signs of RHS, possibly explained by reduced cardiopulmonary reserve due to preexisting cardiovascular diseases. As a result, symptoms linked to impending right heart failure such as syncope, dyspnoea or even cardiac arrest were more likely to be present in elderly patients. This finding is in accordance with the findings of Altinsoy et al. [26] and Islam et al. [22], who both observed that older patients more frequently had a syncope as an initial symptom of acute PE.

## 5. Conclusions

In summary, our analyses confirm chest pain as a favorable prognostic marker in patients with acute PE. The likelihood of chest pain varied significantly based on the patient’s age, while sex appears to have no significant influence. Chest pain was more frequent in young patients who had fewer cardiopulmonary comorbidities and thus presumably better compensatory mechanisms.

Furthermore, our findings support the hypothesis that chest pain in the context of acute PE is due to pleuritic irritation associated with pulmonary infarction as indicated by higher rates of thrombus-associated parenchymal changes on diagnostic CTPA and higher rates of clinically diagnosed pneumonia. In contrast, this study found no association between chest pain and the presence of CAD and increase in troponin levels at presentation, making an ischemic origin unlikely.

## 6. Limitations of the Study

While the presence of chest pain was prospectively assessed in all patients included in PERGO, the quality of chest pain was only documented prospectively starting in May 2018. Thus, this study cannot provide information on pain quality and intensity (e.g., angina or pleuritic thoracic pain). The rate of missing values in baseline characteristics and outcomes in this retrospective study overall was moderately low (see Table A3).

For calculation of algorithms and scores, missing values were considered to be normal. Missing values in the regression variables were handled using complete case analysis (listwise deletion). For the regression analyses, observations with missing values in any variables included in the model were excluded.

Further, all analyses are based on a single-center cohort, thus limiting generalizability of our data.

## Figures and Tables

**Table 1:** Baseline characteristics and outcomes in the study cohort and in patients with and without chest pain.

	Total (n = 858)	Chest Pain (n = 435)	No Chest Pain (n = 423)	*p*-Value
Age [years]	68 (56–78)	66 (51–77)	70 (58–78)	0.001
Sex	♂ 48.0%	♂ 49.7%	♂ 46.3%	0.331
	(412/858)	(216/435)	(196/423)	
	♀ 52.0%	♀ 50.3%	♀ 53.7%	
	(446/858)	(219/435)	(227/423)	
Comorbidities				
Malignancy	22.8% (196/858)	19.1% (83/435)	26.7% (113/423)	0.008
Chronic heart failure	14.2% (122/858)	12.0% (52/435)	16.5% (70/423)	0.054
Coronary artery disease	16.6% (142/858)	17.0% (74/435)	16.1% (68/423)	0.712
Chronic lung disease	17.1% (147/858)	17.0% (74/435)	17.3% (73/423)	0.924
Arterial hypertension	64.6% (554/858)	60.9% (265/435)	68.2% (289/423)	0.023
Atrial fibrillation	10.4% (89/857)	8.0% (35/435)	12.8% (54/422)	0.023
Anemia	36.3% (311/857)	31.5% (137/435)	41.2% (174/422)	0.003
Symptoms of pulmonary embolism				
Unilateral leg swelling	21.0% (180/858)	18.6% (81/435)	23.4% (99/423)	0.085
Unilateral leg pain	19.1% (158/827)	17.3% (72/435)	21.9% (86/423)	0.175
Dyspnea	82.4% (707/858)	82.8% (360/435)	82.0% (347/423)	0.780
Hemoptysis	2.8% (24/858)	4.1% (18/435)	1.4% (6/423)	0.016
Syncope	13.2% (113/858)	7.4% (32/435)	19.1% (81/423)	<0.001
Altered mental status	5.1% (44/858)	2.1% (9/435)	8.3% (35/423)	<0.001
Clinical signs				
Tachycardia: Heart rate >100 bpm	33.4% (281/842)	28.0% (120/428)	38.9% (161/414)	0.001
Hypotension: Systolic blood pressure <90 mmHg	4.9% (40/823)	3.1% (13/423)	6.8% (27/400)	0.014
Hypoxia: spO_2_ < 90%	24.2% (177/732)	17.6% (66/376)	31.2% (111/356)	<0.001
Imaging signs				
Thrombus-associated parenchymal changes on CTPA	35.8% (247/690)	41.8% (148/354)	29.5% (99/336)	0.001
Signs of right heart strain on CTPA/echocardiography	70.0% (575/821)	65.8% (275/418)	74.4% (300/403)	0.007
Laboratory parameters				
Elevated troponin levels	66.7% (540/810)	56.4% (234/415)	77.5% (306/395)	<0.001
Risk stratification according to the ESC 2019 guideline				
Low risk	10.3% (88/858)	15.2% (66/435)	5.2% (22/423)	<0.001
Intermediate-low risk	50.1% (430/858)	53.6% (233/435)	46.6% (197/423)	
Intermediary-high risk	34.5% (296/858)	28.3% (123/435)	40.9% (173/423)	
High risk	5.1% (44/858)	3.0% (13/435)	7.3% (31/423)	
Complications during hospitalization				
Pneumonia	36.0% (309/858)	42.3% (184/435)	29.6% (125/423)	<0.001
Outcome				
PE-related complications	5.9% (51/858)	3.2% (14/435)	8.7% (37/423)	0.001
All-cause mortality	3.3% (28/858)	1.6% (7/435)	5.0% (21/423)	0.006

ESC = European Society of Cardiology; PE = pulmonary embolism, bpm: beats per minute, mmHg: millimeters of mercury, spO_2_: peripheral oxygen saturation, and CTPA: computed tomography pulmonary angiogram.

**Table 2:** Odds for the occurrence of chest pain by age category.

Age Category (Years)	OR [95% CI]
18–34	2.88 [1.69–5.14]
35–59	0.38 [0.20–0.69]
60–79	0.33 [0.18–0.58]
≥80	0.26 [0.13–0.49]

OR = odds ratio; CI = confidence interval.

**Table 3:** Relative frequencies of chest pain, signs of right heart strain, signs of myocardial ischemia and pre-existing conditions by age. Predictive statistics.

Age Category [in Years]	18–34 (n = 66)	35–59 (n = 214)	60–79 (n = 414)	≥80 (n = 164)
Chest pain n = 435	75.6%	49.5%	49.0%	43.3%
Pneumonia n = 309	51.2%	30.9%	35.3%	36.0%
Thrombus-associated parenchymal changes on CTPA n = 247	49.3%	35.6%	34.5%	31.9%
Right heart strain n = 575	48.2%	61.7%	75.9%	77.1%
Troponin elevation n = 540	32.9%	50.3%	72.1%	89.9%
Syncope n = 113	5.8%	10.3%	13.3%	20.1%
Altered mental status n = 44	1.2%	4.1%	3.9%	11.6%
CAD n = 142	0%	6.2%	17.6%	34.8%
CHF n = 122	0%	6.2%	14.0%	31.7%
Malignancy n = 196	9.3%	23.7%	28.5%	14.5%

CAD = coronary artery disease; CHF = chronic heart failure; and CTPA = computed tomography pulmonary angiogram.

**Table 4:** Logistic regression of the occurrence of chest pain for PE-related complications and all-cause mortality in subgroups divided by the median inflection point of 60 years.

	PE-Related Complications		All-Cause Mortality	
	OR [95% CI]	*p*-Value	OR [95% CI]	*p*-Value
Presence of chest pain in subgroup aged <60 years (n = 280)	0.35 [0.08–1.28]	0.011	0.09 [0.01–0.71]	0.004
Presence of chest pain in subgroup aged ≥60 years (n = 578)	0.35 [0.17–0.72]	0.005	0.56 [0.20–1.49]	0.336

PE = pulmonary embolism; OR = odds ratio; and CI = confidence interval.

## Data Availability

The original contributions presented in this study are included in the article/Appendix A. Further inquiries can be directed to the corresponding authors.

## Abbreviations

bpm	Beats per minute
CAD	Coronary artery disease
CHF	Chronic heart failure
CI	Confidence interval
CTPA	Computed tomography pulmonary angiogram
ESC	European Society of Cardiology
hsTnI	High-sensitivity troponin I
hsTnT	High-sensitivity troponin T
IQR	Interquartile range
mmHg	Millimeters of mercury
OR	Odds ratio
PE	Pulmonary embolism
PERGO	Pulmonary Embolism Registry GOettingen
RHS	Right heart strain
RV/LV	Right-to-left ventricular diameter ratio
sPESI	Simplified Pulmonary Embolism Severity Index
spO_2_	Peripheral oxygen saturation

## Appendix A

**Table A1:** Univariable analysis for PE-related complications and all-cause mortality.

	PE-Related Complications		All-Cause Mortality	
	OR [95% CI]	*p*-Value	OR [95% CI]	*p*-Value
Age ≥ 60 years	0.68 [0.38–2.80]	0.376	2.02 [0.69–5.89]	0.198
Female sex	1.34 [0.76–2.39]	0.314	1.07 [0.50–2.27]	0.864
Comorbidities				
Malignancy	0.81 [0.40–1.66]	0.570	3.56 [1.67–7.60]	0.001
Chronic heart failure	3.02 [1.62–5.65]	0.001	1.68 [0.67–4.23]	0.272
Chronic artery disease	2.23 [1.19–4.20]	0.012	1.39 [0.55–3.50]	0.482
Chronic lung disease	1.19 [0.58–2.44]	0.629	1.33 [0.53–3.35]	0.541
Arterial hypertension	3.11 [1.44–6.71]	0.003	3.40 [1.17–9.88]	0.025
Atrial fibrillation	1.67 [0.76–3.67]	0.205	1.93 [0.71–5.21]	0.195
Anemia	3.18 [1.77–5.72]	0.001	3.88 [1.73–8.69]	0.001
Symptoms of pulmonary embolism				
Chest pain	0.35 [0.19–0.65]	0.001	0.31 [0.13–0.74]	0.009
Dyspnea	1.16 [0.53–2.52]	0.711	0.63 [0.26–1.51]	0.300
Hemoptysis	0.68 [0.09–5.15]	0.710	0.00 [0.00–0.00]	0.998
Syncope	2.42 [1.25–4.70]	0.009	1.84 [0.73–4.65]	0.195
Altered mental status	5.55 [2.56–12.00]	0.001	3.29 [1.09–9.94]	0.035
Clinical signs				
Tachycardia: Heart rate >100 bpm	2.50 [1.39–4.49]	0.002	1.18 [0.53–2.61]	0.682
Hypotension: Systolic blood pressure < 90 mmHg	13.64 [6.58–28.27]	0.001	2.56 [0.74–8.90]	0.138
Hypoxia: spO_2_ < 90%	2.59 [1.41–4.77]	0.002	2.16 [0.95–4.89]	0.066
Imaging signs				
Thrombus-associated parenchymal changes	1.34 [0.72–2.47]	0.353	1.36 [0.56–3.27]	0.495
Signs of right heart strain	0.84 [0.46–1.52]	0.561	1.61 [0.64–4.06]	0.312
Laboratory parameters				
Elevated troponin levels	6.53 [2.32–18.36]	0.001	7.18 [1.69–30.52]	0.008

PE: pulmonary embolism, OR = odds ratio; CI = confidence interval, bpm: beats per minute, mmHg: millimeters of mercury, and spO_2_: peripheral oxygen saturation.

**Table A2:** Models of the multivariable logistic regression analysis. Model I: OR of chest pain and PE-related complications/all-cause mortality accounting for prognosis-relevant symptoms of pulmonary artery embolism for the respective endpoint in univariable analyses. Model II: OR of chest pain and PE-related complications/all-cause mortality accounting for coronary artery disease. Model III: OR of chest pain and PE-related complications/all-cause mortality accounting for prognosis-relevant comorbidities for the respective endpoint in univariable analyses. Model IV: OR of chest pain and PE-related complications/all-cause mortality accounting for the risk class according to ESC Guidelines of 2019. Model V: Odds Ratio of chest pain and PE-related complications/all-cause mortality accounting for sex.

**Model I for PE-Related Complications**			**Model I for All-Cause Mortality**		
	**OR [95% CI]**	***p*** **-Value**		**OR [95% CI]**	***p*** **-Value**
Chest pain	0.42 [0.22–0.80]	0.008	Chest pain	0.34 [0.14–0.82]	0.016
Altered mental status	3.83 [1.66–8.34]	0.002	Altered mental status	2.49 [0.81–7.66]	0.111
Syncope	1.44 [0.70–3.01]	0.329			
**Model II for PE-Related Complications**			**Model II for All-Cause Mortality**		
	**OR [95% CI]**	***p*** **-Value**		**OR [95% CI]**	***p*** **-Value**
Chest pain	0.34 [0.18–0.64]	0.001	Chest pain	0.31 [0.13–0.74]	0.008
Coronary artery disease	2.30 [1.22–4.36]	0.010	Coronary artery disease	1.42 [0.56–3.59]	0.456
**Model III for PE-Related Complications**			**Model III for All-Cause Mortality**		
	**OR [95% CI]**	***p*** **-Value**		**OR [95% CI]**	***p*** **-Value**
Chest pain	0.41 [0.22–0.78]	0.007	Chest pain	0.40 [0.16–0.96]	0.039
Chronic heart failure	2.05 [1.02–4.14]	0.045	Malignancy	2.59 [1.15–5.79]	0.021
Coronary artery disease	1.41 [0.69–2.87]	0.351	Arterial hypertension	3.03 [1.03–8.95]	0.045
Arterial hypertension	2.25 [1.01–4.98]	0.046	Anemia	2.56 [1.09–6.02]	0.031
Anemia	2.72 [1.50–4.95]	0.001			
**Model IV for PE-Related Complications**			**Model IV for All-Cause Mortality**		
	**OR [95% CI]**	***p*** **-Value**		**OR [95% CI]**	***p*** **-Value**
Chest pain	0.49 [0.26–0.95]	0.034	Chest pain	0.41 [0.17–0.99]	0.048
Risk class according to ESC 2019	4.10 [2.64–6.36]	0.001	Risk class according to ESC 2019	2.59 [1.51–4.43]	0.001
**Model V for PE-Related Complications**			**Model V for All-Cause Mortality**		
	**OR [95% CI]**	***p*** **-Value**		**OR [95% CI]**	***p*** **-Value**
Chest pain	0.35 [0.18–0.66]	0.001	Chest pain	0.31 [0.13–0.75]	0.009
Female sex	1.30 [0.73–2.33]	0.368	Female sex	1.03 [0.48–2.20]	0.936

OR = odds ratio; CI = confidence interval; PE = pulmonary embolism; and ESC = European Society of Cardiology.

**Table A3:** Missingness table of baseline characteristics and outcomes in the study cohort.

	Total n = 858
Comorbidities	
Malignancy	0/0%
Chronic heart failure	0/0%
Coronary artery disease	0/0%
Chronic lung disease	0/0%
Arterial hypertension	0/0%
Atrial fibrillation	1/0%
Anemia	1/0%
Symptoms of pulmonary embolism	
Unilateral leg swelling	0/0%
Unilateral leg pain	31/4%
Dyspnea	0/0%
Hemoptysis	0/0%
Syncope	0/0%
Altered mental status	0/0%
Clinical signs	
Tachycardia: Heart rate >100 bpm	16/2%
Hypotension: Systolic blood pressure <90 mmHg	35/4%
Hypoxia: spO_2_ < 90%	126/17%
Imaging signs	
Thrombus-associated parenchymal changes on CTPA	168/24%
Signs of right heart strain on CTPA/echocardiography	37/5%
Laboratory parameters	
Elevated troponin levels	66/8%
Complications during hospitalization	
Pneumonia	0/0%
Outcome	
PE-related complications	0/0%
All-cause mortality	0/0%

PE = Pulmonary embolism, bpm: beats per minute, mmHg: millimeters of mercury, spO_2_: peripheral oxygen saturation, and CTPA: computed tomography pulmonary angiogram.
